# The effect of seizure on school attendance among children with epilepsy: a follow-up study at the pediatrics neurology clinic, Tikur Anbessa specialized hospital, Addis Ababa, Ethiopia

**DOI:** 10.1186/s12887-020-02149-y

**Published:** 2020-06-03

**Authors:** Oumer Hassen, Ayalew Beyene

**Affiliations:** Pediatrics and Child Health, Hiwot Fana Specialized University Hospital, Haramaya University, Harar, Ethiopia

**Keywords:** Epilepsy, Epileptic attack; school absenteeism, Childhood epilepsy, Epilepsy in Ethiopia

## Abstract

**Background:**

Epilepsy is the most common chronic neurological disease seen in Pediatrics Neurology Units in many developing countries. It affects negatively on school attendance and academic performance. This study tries to assess the extent and factors contributing school absenteeism among school-aged children and adolescents among epilepsy cases attending at Tikur Anbessa Hospital, Addis Ababa, Ethiopia.

**Methods:**

A hospital based follow-up study was conducted among school-aged children and adolescents with epilepsy between the ages of 7–18 years attending an outpatient Pediatric Neurology Clinic. A sample of consecutive 183 children and adolescents were included in the study full filling criteria of “attended school for at least 6 months in an academic year and walk by themselves with no disability.” The participants (children and their parents/caregivers) gave information concerning the socio-demographic characteristics of the child and the primary caregiver, and review of the child’s presentation and school absenteeism was defined as the average missed days per month over 6 months period and was asked in the questionnaire. Medical charts were thoroughly reviewed on the diagnosis and treatment they received. Study participants characteristics were described using frequency tables and factors associated with school absenteeism were analyzed using logistic regression.

**Results:**

School absenteeism (≥1 days/month) over 6 month period among children aged 7-18 years with epilepsy was 69.4%. Factors which correlated with school absenteeism included female sex (AOR 2.19, 95% CI 1.03–4.84), children with known causes for seizures (AOR 2.51, 95% CI 1.09–5.86), not experiencing seizure at school (AOR 0.39(0.17–0.89) and longer epilepsy duration (AOR 2.36: 1.09, 5.15). The mean age at onset Epilepsy was 4.6 years (±SD = 3.6). One hundred and thirty two (72.1%) had generalized epilepsy, 49(26.8%) had focal epilepsy and the remaining 2(1.1%) had unclassified epilepsy. One hundred and thirty (71.4%) received mono-therapy.

**Conclusion:**

Experience of school absenteeism reported by over two thirds of children aged 7–18 years with epilepsy attending an outpatient epilepsy clinic in Ethiopia. Children with known seizure should be followed regularly, and compensation for missed school has to be organized.

## Background

Epilepsy is a disorder of the brain characterized by an enduring predisposition to generate seizures and by the neurobiological, cognitive, psychological, and social consequences of this condition and clinical diagnosis of epilepsy usually requires the occurrence of at least 1 unprovoked epileptic seizure with either a second such seizure or enough EEG and clinical information to convincingly demonstrate an enduring predisposition to develop recurrences. For epidemiologic and commonly for clinical purposes, epilepsy is considered to be present when 2 or more unprovoked seizures occur in a time frame of longer than 24 h [1].

Epilepsy is the most common chronic neurological disease seen in Pediatrics Neurology Units in developing countries [2] and has prevalence of 5–10/1000 people in developing countries [3]. A prior study from our center, in 2013 reported that seizure disorders were found in 45.6% of all children attending our outpatient neurology clinic. Of these, 28.8% of seizure disorders (30 of 104) were of unknown cause and 71.2% (74 of 104) were secondary to other disorders [4].

Children with epilepsy are prone to educational underachievement as a result of co-morbid learning and behavioral problems. Other factors that may contribute to poor school performance amongst epilepsy patients may include overprotective parental attitudes, a lack of academic motivation, and low self-esteem. The impact of epilepsy on school attendance may also contribute to the academic difficulties of children with epilepsy.

Although most studies addressing academic performance in school-aged children with epilepsy are focused on academic achievement and quality of life of children with epilepsy, only a few address the impact of epilepsy on school attendance. One prospective study from a specialty pediatric epilepsy clinic in Brazilfound that 88% of patients in the study missed at least 1 day of school due to seizures. Nearly half of parents (46%) believed that if the child had a seizure at school he/she should leave school immediately [5].

In another similar study there were several variables that may be related to the school absenteeism, such epilepsy type, severity, age at onset, but also the beliefs of parents, teachers and school friends, who often stigmatize children with epilepsy, believing that such children present more behavioral problems than healthy children of similar intellect [6].

In Ethiopia, a recent survey of knowledge, attitude, and practice of teachers towards people with epilepsy (PWE) found that 90 % knew epilepsy as a disease, 51.3% indicated that the source of information was acquaintances with PWE and 28.6%had a student with epilepsy in class. Although 89.2% of the teachers would allow PWE into their class, the majority (76.7%) preferred that the epilepsy be cured or controlled before attendance because they were perceived insane more than infectious explaining [7].

This study assesses the effect of childhood epilepsy on school attendance and determines the reasons for absenteeism.

## Methods

### Study setting

The study was conducted in the outpatient pediatric neurology clinic in Addis Ababa, Ethiopia at Tikur Anbessa Specialized Hospital (TASH). It is the largest referral hospital in Ethiopia with a number of specialties and subspecialty units. The hospital has totally around 600 admission beds and serves about 250,000 outpatients annually.

The TASH pediatric neurology clinic visited by approximately 600 children and adolescents aged from 0 to 18 years per month. Clinically stable patients with epilepsy who have with good seizure control are appointed to come every 2–3 months.

### Design and study population

This cross-sectional study was conducted at the PNC in TASH between April and July 2018. All school aged children and adolescents aged 7–18 years, attending the clinic with their primary caretaker during the study period that had a 6 month or longer history of epilepsy were invited to participate. Consent was obtained from a parent or guardian on behalf of any participants under the age of 16. Children with physical disability who were unable walk independently, and those in whom parental consent was not obtained were excluded. Consented study participants were included in the study consecutively until the sample size was attained.

### Data collection and analysis

A structured study questionnaire was administered among families of epileptic case by 4 pediatric residents, under the supervision of the attending neurologist. We defined school absenteeism as the average missed school days per month (≥1 days/month) over 6 month period from January 1, 2018 until the date of completion of the sample size. Further to understand the reasons for missing school days, it was specifically asked if the missed days due to seizure.

In this study, we consider symptomatic seizures as seizure/epilepsy that follows an injury (head injury, CNS infection, stroke, brain tumor, and surgery) to the brain known to be capable of causing epilepsy [1]. And seizure control as *complete* if children were seizure-free for > 6 months, as *partial* if there was a > 50% reduction in seizure and as *poor* if they had one or more seizure per month over of the last 6 months despite trials of at least two different AEDs at optimum doses alone or in combination with adequate compliance [8].

Data analysis was done using the Statistical Package for Social Sciences (SPSS) version 22. Variables potentially associated with absenteeism were assessed by crud analysis, and those variables with significant association in the crude analysis were considered in the adjusted analysis. Odds ratios with the 95% confidence interval have been considered to report the results. For all statistical analysis a *p* value of < 0.05 is considered statistically significant.

## Results

### Socio-demographic characteristics

This study included 183 children with epilepsy and demographics are shown in Table 1. More than half (55.7%) were male and most (72.7%) were in the age group of 7 to 12 years. One hundred twenty-three (67.2%) of 183 attended primary school. Fifty six (30.6%) were attending nursery school which is not age appropriate, and 84(45.9%) repeated at least one grade.

**Table 1:** Socio demographic characteristics of school age children and adolescents and primary caregivers with seizure disorder attending follow up at PNC in TASH between April and July, 2018

Variables	Frequency	Percentage
Age groups in year		
7–12	133	72.7
13–18	50	27.3
Mean/±SD =10.47/2.9		
Sex		
Male	102	55.7
Female	81	44.3
Address		
Addis Ababa	118	64.5
Out of Addis Ababa	65	35.5
Childs level of education		
Nursery	56	30.6
Primary school	123	67.2
Secondary school	4	2.2
Repeated grade		
Never repeated	99	54.1
Repeated	84	45.9
Marital status of primary care giver		
Single	9	4.9
Married	148	80.9
Divorced	15	8.2
Widowed	11	6.0
Educational status of primary care giver *n* = 181		
Less than high school	73	40.3
High school	39	21.5
Collage level and above	69	38.1
Family monthly income (*n* = 140)		
< 1500 birr (50 US $)	65	46.4
> 1500 birr	75	53.6

The primary caregiver for the child was reported to be mothers in 71(38.8%), fathers in 34 (18.6%) and both parents equally in 68 (37.2%) of cases. The majority of the primary caregivers were married (80.9%).One hundred eight (59.9%) primary caregivers completed secondary school education or greater. Only 140 (76.5%) of the care givers disclosed their income, among whom only 75(53.6%) earned > 1500 Birr/month (Table 1).

### Seizure characteristics

The mean age at onset was 4.6 years (±SD = 3.6) and mean duration of epilepsy was 5.7 years (±SD = 3.5). One hundred and thirty two (72.1%) had generalized epilepsy, 49(26.8%) had focal epilepsy and the remaining 2(1.1%) had unclassified epilepsy (Table 3).

Most patients 104(56.8%) had seizures once per month or less. One hundred and thirty (71.4%) received mono-therapy. The available drugs are sodium valproate, carbamazepine, phenobarbital, phenytoin, and clonazepam. We also use Lamotrigine and Levetiracetam in very few patients as it is less available and expensive. Of the total 183 study subjects About 44(24.0%) had a known cause for epilepsy; while the rest 139(76.0%) do not have a known cause. Complete seizure control was observed in 92(50.3%), partial control in 57(31.1%), poor control in 33(18.1%) and not documented in one patients (Table 2).

**Table 2:** Seizure related information of school age children and adolescents with seizure disorder attending follow up at PNC in TASH between April and July, 2018

Variables	Frequency	Percentage
Age at onset		
< 1 year	36	19.7
1–5 year	76	41.5
> 5 year	71	38.8
Mean/±SD = 4.6/3.6		
Duration of epilepsy in years		
< 5	70	38.3
≥ 5	113	61.7
Mean/±SD = 5.7/3.5		
Seizure type		
Focal onset	49	26.8
Generalized onset	132	72.1
Unknown onset	2	1.1
Seizure frequency		
≤1 per month	104	56.8
> 1per month	79	43.2
Type of therapy (*n* = 182)^a^		
Monotherapy	130	71.4
Monotherapy& have side effects	3	1.6
Poly-therapy	46	25.3
Poly-therapy & have side effects	3	1.6
Comorbidity		
Yes	47	25.7
No	136	74.3
Symptomatic epilepsy with a known cause		
Yes	44	24.0
No	139	76.0
Seizure control		
Complete seizure control	92	50.3
Partial seizure control	57	31.1
Poor seizure control	33	18.0
Not documented	1	0.5

^a^AEDs used were Sodium Valproate, Carbamazepine, Phenobarbital, Phenytoin, and Clonazepam. We also use Lamotrigine and Levetiracetam in very few patients as it is less available and expensive

### School absenteeism

One hundred and twenty seven of 183(69.4%) children were reported to miss school days due to seizures and most (90) missed between 1 to 10 days, among children who missed school days 25.9% of children missed more than 50% of school days (Table 3) and only 2.2% were pulled out of school during that year (2018). Reasons given by caregivers for missed school included medical appointments ((80.3%), seizure occurring prior to school day (50.4%), and seizure occurring at school (21.3%). The primary care givers allowed 19.2% of their children to miss school days even when there is no illness, predominantly due to concern that the child may have a seizure in school in 75% of cases.

**Table 3:** Effect of seizure on school attendance of school age children and adolescents with seizure disorder attending follow up at PNC in TASH between April and July, 2018

Variables	Frequency	Percentage
Ever miss a school day because of seizure		
Yes	127	69.4
No	56	30.6
Number of days missed (*n* = 127)		
< 10 days	90	70.9
10–30 days	33	25.9
Pulled out of school	4	3.2
Mean/±SD = 12.6/31		
% of days missed (*n* = 127)		
< 50%	90	70.9
≥50%	37	29.1
Reasons for missing school due to seizure (*n* = 127)		
Had a seizure before school	64	50.4
Had a seizure at school and needed to go home	27	21.3
Had a medical appointment	102	80.3
Had a test scheduled	1	0.8
Other	1	0.8
Had Seizure at school(*n* = 183)	88	48.1
went home before the end of classesafter seizure in school(*n* = 88)	81	9
Should go home from schools immediately for reasons of seizure		
Should go home immediately	58	31.7
Can stay at school if well	125	68.3
Teacher suggested stay at home as the child had seizure experience		
Yes	31	17.0
No	151	83.0
Allowed child to miss a day of school even if the child is not sick		
Yes	35	19.2
No	145	80.8
Reasons for allowing miss a class for seizure(*n* = 35)		
Fear of seizure at school	27	75.0
No particular reason	3	8.3
Other	6	16.7
Guardians disclose child’s epilepsy status to a teacher(*n* = 181)		
Yes	159	86.9
Guardians disclose child’s epilepsy status to a peer		
Yes	105	57.4
No	78	42.6

Caregivers reported that teachers had expressed apprehension to them about their child’s epilepsy in 71(38.2%) of the cases. The teachers were mainly concerned because they fear that other students could be disturbed and the school performance of affected children may decline. In 17% of cases, teachers requested that children with epilepsy should stay at home. Predictors for school absenteeism are shown in Table 4. Older age group, female sex, epilepsy duration ≥5 years, repeating one or more grades, prior seizure at school, higher seizure frequency, longer seizure duration and disclosing seizure to teachers, were associated with school absenteeism. Further analysis by multivariable analysis showed female students (AOR 2.19: 1.03, 4.84), having a known cause for epilepsy (AOR 2.51: 1.09, 5.86), and having longer duration of seizure (seizure experience for more than 5 years) (AOR 2.36: 1.09, 5.15) were identified as independent determinants of poor school attendance.

**Table 4:** Factors associated with school absenteeism of school age children and adolescents with seizure disorder attending follow up at PNC in TASH between April and July, 2018

Variables	School Absenteeism		COR(95%CI)	AOD (95% CI)
	No (%)	Yes (%)		
Age groups				
7–12 years	47	86	2.49(1.1–5.6)	2.01(0.83–5.24)
13–18 years	9	41		
Sex				
Male	39	63	2.33(1.2–4.6)	2.19(1.03–4.84)
Female	17	64		
Family size				
< 5 members	24	57	0.89(0.45–1.70)	
≥5 members	32	70		
Duration of seizure				
< 5	9	8	3.12(1.64–6.05)	2.36(1.09–5.15)
≥5	47	119		
Repeated grade				
Never repeated	38	61	2.28(1.19–4.49)	1.57(0.73–3.42)
Repeated	18	66		
Marital status of primary care giver				
Single	28	7	0.51 (0.19–1.19)	
Married	98	50		
Educational status of primary care giver				
< high school	16	57	1.0 (0.5–1.9)	
High school	12	27	1.31 (0.2–27.4)	
College +	28	41		
Seizure frequency				
= < 1 month	39	65	2.18(1.13–4.34)	1.55(0.69–3.55)
> 1 month	17	62		
Seizure at school				
No	42	53	4.19(2.1–8.4)	0.39(0.17–0.89)
Yes	14	74		
Symptomatic seizure				
No	35	104	2.71(1.33–1.55)	2.51(1.09–5.86)
Yes	21	23		
Guardians disclosed epilepsy status to teacher				
No	13	11	3.19(1.33–7.6)	0.40(0.13–1.18)
Yes	43	116		

## Discussion

In this study, school absenteeism among children aged 7–18 years at PNC follow up were 69.4%. Poor attendance is less in this study compared to the research in Brazil (88%) but higher than the study in Sera lion (50%) and a study by CDC (36%) [5, 9, 10]. The differences in estimates poor school attendance could be attributed to the difference in sample size, the demographic characteristics, seizure duration, and the varying definition of poor attendance.

More children with epilepsy within the age group of 13–18 years (82%) missed school. The higher proportion of absenteeism in this age group may reflect the difficulty of coping with epilepsy among older children due to possible longer duration of the disease since onset and fear of dealing with the stigma associated with the illness.

Totally four children (3.2%) ceased attending school in our study is fewer than reported in a study done in Serra lion which was about 20%. Education is very important for all children and especially for children with epilepsy as it could facilitate adaptive functionality and better integration in the society. Therefore, cessation of education should not an option in children with epilepsy rather allowing children to come to clinic after school after medical appointments and improved seizure control might help children decrease absenteeism.

In this study, there were statistically significant association between female sex and missed school days. More female (79%) than male (63%) children with epilepsy missed school days. Female children with epilepsy were 2.2 times more likely to miss school days than their male counterparts. In developing countries like Ethiopia where enrollment and retention of female children at school is poor, this result is expected. In addition, female children with epilepsy are at increased risk of developing depression due to the illness which could perhaps affect school attendance negatively. The finding of this study shows the need to reach out for female children with epilepsy even more than the male children. Stronger social support to boost the confidence and better function of children with epilepsy are required.

More number of care givers has disclosed the condition of the children to the teachers (86.9%). As teachers could support children with epilepsy, it is encouraging to know that most of the careers willingly disclose the status of the children to their teachers. The preference of the teachers to let the children stay at home till their seizure is cured before coming to school can be the reason for poor attendance [7]. It is important to equip teachers with the knowledge and resources required to handle children with epilepsy to avoid more number of missing days. Furthermore, teachers and care takers could plan ways to make up for missed days of school to improve attendance of the children.

Having symptomatic seizure and longer duration of seizure identified as the independent determinants of missing school. This could be associated with the illness and potential fear of stigma associated with having the illness. Children who experienced seizure at school are less likely to miss school than those who never had seizure at school. This could perhaps be due to a good collaboration of the care takers and teachers in reassuring the children with seizure.

Greater proportions of primary caregivers of the children were married (80.9%) and more than half of had attended high school or higher education (59.6%) and have a secured monthly income (68.2%). These characteristics of careers are expected to affect school attendance of children with epilepsy positively. Though, more proportion of children raised by parents of lower education level missed school than those raised by parents of higher education level, our findings fail to demonstrate a statistically significant association between family size, marital status of parents, socioeconomic status and educational status of parent’s with school absenteeism.

As shown in our results despite the absence of any illness the parents allowed 19.2% of their children to miss school days. Excessive fear and concerns of care takers of children with epilepsy need to be tackled through reassurance and education to improve school attendance.

More children who repeated grade (78.6%) missed more school days than those who never repeated their grade (61.6%). There is a statistically significant association between repeating grade and poor attendance. A possible inference from this is that repeating a grade could negatively affect school attendance. Children with epilepsy are likely to have associated learning difficulties, therefore each child learning abilities need to be assessed before commencement and during the learning process. Therefore, the schools need to identify trained personnel who could identify learning challenges of the child and who could suggest potential measures to be taken to improve progress of learning of the children with epilepsy to avoid repeating classes. There is a need for collaborative action by clinicians, teachers, care takers and school administration to improve attendance. Facilitating a regular neuropsychological assessment, continuous follow up and after school clinic will help children in decreasing absenteeism. Training of the teachers and care takers on how to deal on apprehension is necessary. The importance attendance and performance at school for better integration of the children in the society should be emphasized. Special support group should be established in schools to cater the needs and limitations of children with epilepsy.

Our study has potential limitations. This includes the study was conducted in one tertiary hospital which could result in an under or over estimation of the actual burden of the problem. The questioner was also not validated and there may variables that may affect school attendance. Income disclosure among caregivers was limited for further statistical analysis.

## Conclusion

This study identified that school absenteeism is very common among children aged 7–18 years at PNC follow up (69.4%). It was learnt that experience of seizure has significant effect on children’s school attendance. Females, seizure duration for more than 5 years, symptomatic epilepsy are significantly associated with school absenteeism.

## Data Availability

The datasets generated or analyzed during the current study are not publicly available due to intuitional policy but are available from corresponding author on reasonable request.

## Abbreviations

ADHD: Attention Deficit Hyperactivity Disorder
AOR: Adjusted Odds Ratio
CI: Confidence Interval
CWE: Children with Epilepsy
CNS: Central Nervous System
CSHCN: Children with Special Health Care Needs
EEG: Electroencephalography
FMoH: Federal Ministry of Health
ILAE: International League against Epilepsy
OR: Odds Ratio
MDG: Millennium Development Goals
NCDs: Non-Communicable Diseases
PNC: Pediatric Neurology Clinic
SPSS: Statistical Package for Social Sciences
TASH: TikurAnbessa Specialized Hospital
WHO: World Health Organization
SSNPR: Southern Nations, Nationalities, and Peoples’ Region
